# Association of the systemic immune-inflammation index with all-cause and cardiovascular mortality in individuals with rheumatoid arthritis

**DOI:** 10.1038/s41598-024-66152-4

**Published:** 2024-07-02

**Authors:** Xiaoshuang Yin, Yu Zhang, Jinmei Zou, Jing Yang

**Affiliations:** Department of Immunology, Mianyang Central Hospital, School of Medicine, University of Electronic Science and Technology of China, Mianyang, Sichuan China

**Keywords:** Systemic immune-inflammation index, Mortality, Rheumatoid arthritis, NHANES, Biomarker, Rheumatic diseases, Rheumatology

## Abstract

The systemic immune-inflammation index (SII), a metric reflecting systemic inflammatory response and immune activation, remains underexplored concerning its correlation with mortality among rheumatoid arthritis (RA) patients. This study aimed to delineate the association between SII and both all-cause and cardiovascular mortality within the cohort of American adults diagnosed with RA, utilizing data from the National Health and Nutrition Examination Survey (NHANES) spanning 1999 to 2018. The investigation extracted data from NHANES cycles between 1999 and 2018, identifying RA patients through questionnaire responses. The SII was computed based on complete blood counts, employing the formula: (platelets × neutrophils) / lymphocytes. The optimal SII cutoff value for significant survival outcomes was determined using maximally selected rank statistics. Multivariable Cox proportional hazards models assessed the relationship between SII levels and mortality (all-cause and cardiovascular) among RA patients, with subgroup analyses examining potential modifications by clinical confounders. Additionally, restricted cubic spline (RCS) analyses were conducted to explore the linearity of the SII-mortality association. The study encompassed 2070 American adults with RA, among whom 287 exhibited a higher SII (≥ 919.75) and 1783 a lower SII (< 919.75). Over a median follow-up duration of 108 months, 602 participants died. After adjustments for demographic, socioeconomic, and lifestyle variables, a higher SII was associated with a 1.48-fold increased risk of all-cause mortality (hazard ratio [HR] = 1.48, 95% confidence interval [CI] 1.21–1.81, *P* < 0.001) and a 1.51-fold increased risk of cardiovascular mortality (HR = 1.51, 95% CI 1.04–2.18, *P* = 0.030) compared to a lower SII. Kaplan–Meier analyses corroborated significantly reduced survival rates within the higher SII cohort for both all-cause and cardiovascular mortality (*P*_all-cause mortality_ < 0.0001 and *P*_cardiovascular mortality_ = 0.0004). RCS analyses confirmed a positive nonlinear relationship between SII and mortality rates. In conclusion, the SII offers a straightforward indicator of the equilibrium between detrimental innate inflammation and beneficial adaptive immunity. Our investigation, utilizing a comprehensive and nationally representative sample, reveals that elevated SII levels independently forecast a greater risk of mortality from all causes, as well as cardiovascular-specific mortality, in individuals suffering from RA. These insights underscore the clinical relevance of the SII as an affordable and readily accessible biomarker. Its incorporation into regular clinical practice could significantly enhance the precision of risk assessment and forecasting for patients with RA, facilitating more tailored and effective management strategies. Specifically, patients with high SII levels could be identified for more stringent cardiovascular risk management, including closer monitoring, lifestyle interventions, and aggressive pharmacological treatments to mitigate their increased risk of mortality.

## Introduction

Rheumatoid arthritis (RA) constitutes a chronic, systemic inflammatory autoimmune disorder, leading to joint damage and subsequent disability^1^. It represents a significant public health issue globally, with the prevalence estimated at 17.6 million individuals worldwide in 2020, marking a 14.1% increase since 1990. Future projections anticipate a continued rise in the RA burden, expecting the global affected population to reach 31.7 million by 2050^2^. Patients with RA experience a diminished life expectancy, primarily due to cardiovascular disease, the predominant cause of premature death in this group^3,4^. The elucidation of factors that influence the incidence and mortality of RA is vital for the development of effective management and intervention strategies. The persistent inflammation characteristic of RA not only plays a pivotal role in its pathogenesis and progression but is also implicated in the elevated risk of cardiovascular mortality associated with the disease^4,5^. Thus, inflammatory markers may serve a prognostic role in individuals with RA.

An ideal prognostic scoring system should provide easily identifiable prognostic indicators at the time of diagnosis and remain cost-effective for clinical use, thereby ensuring its accessibility and affordability for broad application. The Systemic Immune-Inflammation Index (SII), a measure of systemic inflammatory response, has been established as a prognostic factor for elderly patients with tumors of the digestive system^6,7^. It is derived from a formula incorporating levels of specific immune system markers obtained from complete blood counts, specifically, (platelets × neutrophils) / lymphocytes^7^. Traditionally, SII has been utilized as a mortality predictor for patients with various tumors^6,7,34,36^, cerebrovascular diseases^8,9,11^, and cardiovascular conditions^9,10,12^. The scope of SII's application has been broadening, with recent research indicating its potential in forecasting disease severity and monitoring the efficacy of treatments^13–17^. Furthermore, evidence supports the utility of SII in evaluating the severity and progression in patients with RA^18–20^. Another investigation revealed its predictive value for RA risk among U.S. adults^5^. Elevated SII levels indicate a perpetual state of immune activation, leading to sustained inflammation in joints and other tissues^21,22^. This persistent inflammatory response can result in the degradation of cartilage and bone, manifesting as pain and restricted mobility. These observations underscore the potential of SII as a valuable instrument for tracking inflammation and assessing disease activity in RA patients.

As of now, the National Health and Nutrition Examination Survey (NHANES) database has not been utilized by researchers to investigate the correlation between the SII and mortality risk among patients with RA. Given the critical need for prognostic markers that are both affordable and accessible for RA, our study aimed to examine the link between SII levels and the risk of mortality from all causes, as well as cardiovascular-specific mortality. We posited that elevated SII levels would correlate with increased mortality rates. Our analysis was conducted on a comprehensive and nationally representative cohort of American adults diagnosed with RA, leveraging data from the NHANES database^23^.

## Methods

### Study design and population

The NHANES is a continuous initiative led by the Centers for Disease Control and Prevention (CDC) in the United States, aiming to evaluate the health and nutritional status of American adults and children. This comprehensive survey integrates interviews, physical examinations, and laboratory testing. Detailed methodologies are available on the official CDC website (http://www.cdc.gov/nchs/nhanes.htm, accessed on 1 January 2024). The original survey protocol was subject to thorough ethical review and received the approval of the CDC's Institutional Review Board. All participants provided their informed consent through the signing of consent forms before participating in the study. Our current study, which utilizes data from this approved survey, underwent review by our center's Institutional Review Board. Upon review, it was determined that our study qualified for an exemption category, indicating it adhered to the requisite ethical standards and regulations, thereby negating the need for further ethical approval.

**Figure 1:**
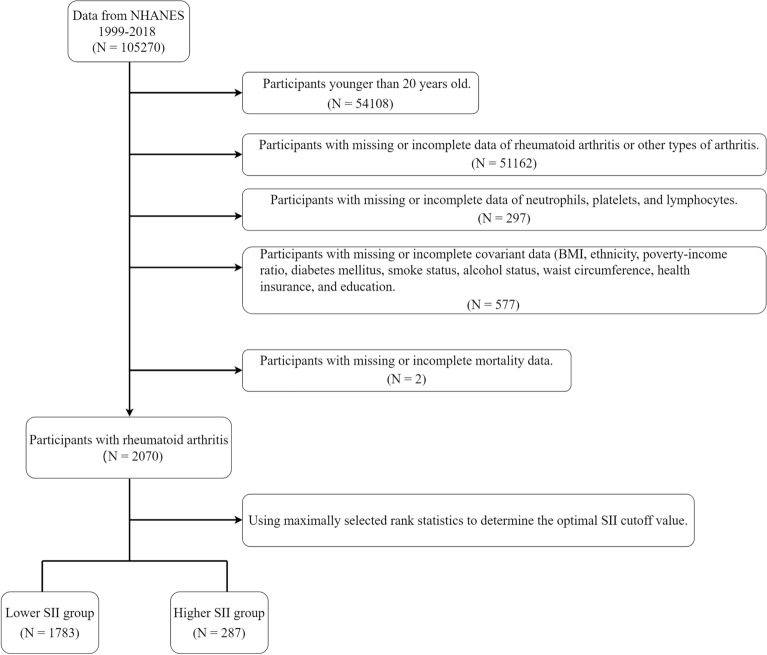
Flow diagram depicting the inclusion and exclusion of participants in the present study.

This prospective cohort study analyzed data from the continuous NHANES spanning from 1999 to 2018. We excluded individuals under the age of 20, those with incomplete data on complete blood count parameters, participants without specific information on RA or other arthritis forms, and individuals lacking essential covariate data. The inclusion and exclusion process of participants is illustrated in Fig. 1.

### Definition of primary variates

Arthritis diagnoses were ascertained through a self-reported questionnaire (MCQ160a), wherein participants were queried, “Has a doctor or other health professional ever told you that you have arthritis?” allowing for responses of either “Yes” or “No.” For those indicating a positive diagnosis, further inquiries were made to identify the specific type of arthritis. The response options provided were “Rheumatoid arthritis,” “Osteoarthritis,” “Psoriatic arthritis,” “Other,” “Refused,” and “Do not know,” to enable the differentiation among various arthritis forms.

The SII was calculated from an automated complete blood count sample using the formula: (platelets × neutrophils) / lymphocytes. The main outcomes of interest were all-cause and cardiovascular mortality, which were determined through mortality-linked files (MLFs) that recorded deaths from any cause. These mortality records were obtained from the National Death Index (NDI) database, available at https://www.cdc.gov/nchs/datalinkage/mortality-public.htm. The duration of follow-up for each participant was measured from the date they participated in the study until either their date of death or December 31, 2019, whichever came first. This end date corresponds to the most recent update of the NDI database.

### Assessment of covariates

The study considered demographic variables such as age, sex, and ethnicity. Socioeconomic factors were represented by educational level and the poverty-income ratio (PIR). Lifestyle and health-related characteristics included body mass index (BMI), smoking habits, health insurance coverage, alcohol consumption, and waist circumference. Medical conditions accounted for included diabetes, hyperlipidemia, and hypertension. Laboratory measurements encompassed red blood cell (RBC) count, white blood cell (WBC) count, neutrophils, platelets, and lymphocytes. The multivariable analyses were progressively adjusted for these factors, starting with demographic and socioeconomic factors, followed by lifestyle and health-related factors, medical conditions, and laboratory measurements. The strategy for handling missing data on covariates was listwise deletion.

### Statistical analysis

**Figure 2:**
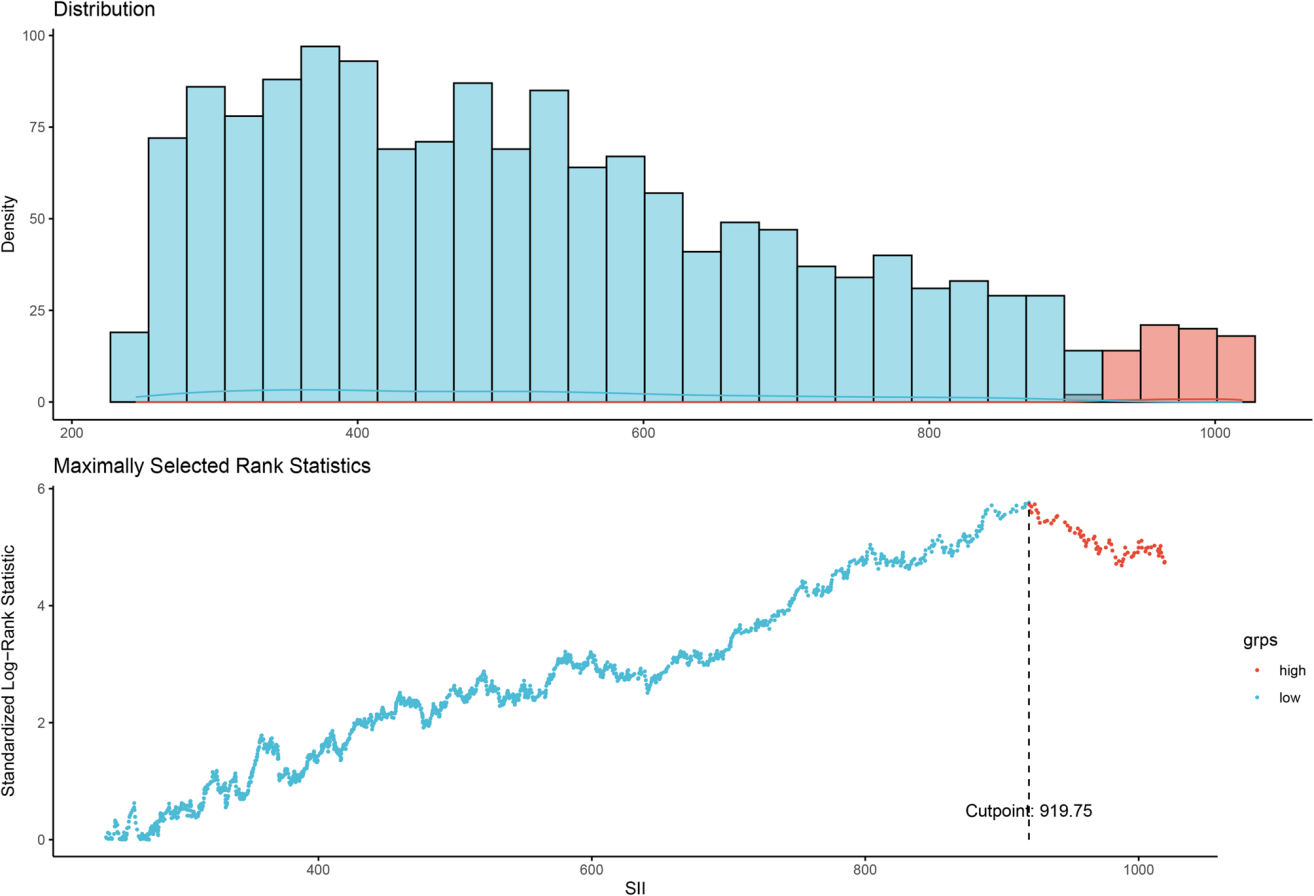
Identification of the SII threshold through the use of maximally selected rank statistics. Standardized Log-Rank Statistic was utilized in the calculation.

Baseline characteristics were presented as means (with standard deviations) for continuous variables and frequencies (with percentages) for categorical variables. The presence of RA or differences across SII categories were evaluated using chi-squared tests for categorical variables and t-tests for continuous ones. We utilized maximally selected rank statistics to determine the optimal cut-off point for the SII in predicting all-cause and cardiovascular mortality. This non-parametric method identifies the threshold that maximizes the separation between high and low-risk groups concerning the outcome of interest, providing a robust and clinically relevant marker for risk stratification. The optimal cutoff point for SII, set at 919.75, was identified for its predictive value in mortality (illustrated in Fig. 2). To explore the link between the SII and both all-cause and cardiovascular mortality among RA patients, Cox proportional hazards models were deployed. These models were structured to include progressively comprehensive adjustments for confounding factors. The initial model (Model 1) applied no adjustments. Model 2 incorporated adjustments for age, sex, ethnicity, and educational level. Model 3 further adjusted for a broader set of variables, including age, sex, ethnicity, educational level, waist circumference, PIR, smoking habits, and health insurance coverage. Survival probabilities across various SII thresholds were depicted through Kaplan–Meier curves. Additionally, subgroup analyses were conducted to explore potential modifications in effect by key clinical variables. The association between SII as a continuous variable and mortality outcomes was illustrated using a restricted cubic spline (RCS) model. All statistical analyses were conducted in R version 4.3.2 (R Foundation for Statistical Computing, Vienna, Austria; http://www.R-project.org), and significance was determined at a two-sided *P*-value of less than 0.05.

### Ethical approval

The initial survey methodology was subjected to thorough ethical examination and was sanctioned by the CDC's Institutional Review Board. The research adhered to the applicable local laws and organizational norms. The legal guardians or next of kin of the participants provided written consent for involvement in the study.

## Results

### Study population

In a cohort of 2070 individuals diagnosed with RA, 287 participants were categorized within the higher SII group (SII ≥ 919.75), while 1783 fell into the lower SII category (SII < 919.75). A greater proportion of participants aged over 60 were observed in the higher SII group (66.20%) compared to the lower SII group (56.98%). Additionally, a higher percentage of White individuals was noted in the higher SII group (51.22%) relative to the lower SII group (42.29%). No significant differences were found between the two SII groups concerning sex, educational levels, poverty-income ratio (PIR), health insurance coverage, smoking habits, alcohol consumption, hypertension, diabetes mellitus, hyperlipidemia, BMI, waist circumference, and RBC count. The average SII was markedly higher in the higher SII group (1155.55) in comparison to the lower SII group (456.75), signifying that SII levels correlate with age and ethnicity but are independent of socioeconomic, lifestyle, and additional health-related factors. Significant disparities were also observed between the higher and lower SII groups in terms of WBC count, neutrophils, platelets, lymphocytes, all-cause mortality, and cardiovascular mortality. These findings are elaborated in Table 1.

**Table 1:** Baseline characteristics of individuals with RA between lower SII group and higher SII group.

Variable	Total (n = 2070)	Lower SII (n = 1783)	Higher SII (n = 287)	*P* value
Age, y, n (%)				0.007
20–39	172 (8.31)	148 (8.30)	24 (8.36)	
40–59	692 (33.43)	619 (34.72)	73 (25.44)	
> 60	1206 (58.26)	1016 (56.98)	190 (66.20)	
Sex, n (%)				0.31
Male	888 (42.9)	757 (42.46)	131 (45.64)	
Female	1182 (57.1)	1026 (57.54)	156 (54.36)	
Ethnicity, n (%)				0.007
White	901 (43.53)	754 (42.29)	147 (51.22)	
Black	590 (28.50)	531 (29.78)	59 (20.56)	
Mexican American	323 (15.60)	279 (15.65)	44 (15.33)	
Others	256 (12.37)	219 (12.28)	37 (12.89)	
Education, n (%)				0.72
Under high school	736 (35.56)	632 (35.45)	104 (36.24)	
High school or equivalent	505 (24.40)	431 (24.17)	74 (25.78)	
College graduate or above	829 (40.05)	720 (40.38)	109 (37.98)	
PIR, n (%)				0.37
0–1.29	817 (39.47)	699 (39.20)	118 (41.11)	
1.30–3.49	776 (37.49)	664 (37.24)	112 (39.02)	
> 3.50	477 (23.04)	420 (23.56)	57 (19.86)	
Health insurance, n (%)	1810 (87.44)	1557 (87.32)	253 (88.15)	0.69
Smoking status, n (%)				0.67
Never	892 (43.09)	774 (43.41)	118 (41.11)	
Current	515 (24.88)	438 (24.57)	77 (26.83)	
Former	663 (32.03)	571 (32.02)	92 (32.06)	
Alcohol use, n (%)				0.92
Never	338 (16.33)	292 (16.38)	46 (16.03)	
Mild	638 (30.82)	555 (31.13)	83 (28.92)	
Moderate	248 (11.98)	214 (12.00)	34 (11.85)	
Heavy	294 (14.20)	250 (14.02)	44 (15.33)	
Former	552 (26.67)	472 (26.47)	80 (27.87)	
Hypertension, n (%)	1359 (65.65)	1157 (64.89)	202 (70.38)	0.07
Diabetes mellitus, n (%)	500 (24.15)	434 (24.34)	66 (23.00)	0.62
Hyperlipidemia, n (%)	1668 (80.58)	1444 (80.99)	224 (78.05)	0.24
BMI group, kg/m^2^, n (%)				0.10
< 25	455 (21.98)	378 (21.20)	77 (26.83)	
25–30	646 (31.21)	563 (31.58)	83 (28.92)	
> 30	969 (46.81)	842 (47.22)	127 (44.25)	
Waist circumference, cm	102.3 (92.9–113.2)	102.3 (93.0–113.0)	102.0 (92.5–115.1)	0.56
RBC, 10^9^/L	4.58 (4.23–4.89)	4.58 (4.24–4.88)	4.58 (4.22–4.91)	0.93
WBC, 10^9^/L	6.9 (5.7–8.5)	6.7 (5.6–8.1)	8.6 (7.1–10.6)	< 0.001
Neutrophils, 10^9^/L	4.1 (3.1–5.2)	3.8 (3–4.8)	6.3 (5.1–7.8)	< 0.001
Platelets, 10^9^/L	244 (205–294)	238 (200–282)	295 (253–369)	< 0.001
Lymphocyte, 10^9^/L	2.0 (1.6–2.5)	2.0 (1.6–2.6)	1.5 (1.1–2.0)	< 0.001
SII	501.95 (345.00–725.00)	456.75 (325.57–606.10)	1155.55 (1014.55–1457.00)	< 0.001
All-cause mortality, n (%)	602	476 (26.70)	126 (43.90)	< 0.001
Cardiovascular mortality, n (%)	169	132 (7.40)	37 (12.89)	< 0.001

RA: Rheumatoid arthritis; SII: Systemic immune-inflammation index; PIR: Poverty income ratio; BMI: Body mass index; RBC: Red blood cell; WBC: White blood cell.

### Association between SII and all-cause and cardiovascular mortality

During a median follow-up period of 108 months, 602 individuals with RA passed away, of which 169 deaths were attributed to cardiovascular disease. Among these, the group with higher SII saw 126 (43.90%) deaths from all causes and 37 (12.89%) from cardiovascular causes. In comparison, the group with lower SII experienced fewer deaths overall. In the initial unadjusted analysis (Model 1), a higher SII, analyzed as a continuous variable, was significantly linked with an increased risk of mortality from all causes (hazard ratio [HR] = 1.03, 95% confidence interval [CI] 1.01–1.05, *P* < 0.001) and cardiovascular causes (HR = 1.03, 95% CI 1.01–1.06, *P* < 0.001). When assessed categorically, being in the higher SII group was associated with a 1.80-fold increase in all-cause mortality risk (HR = 1.80, 95% CI 1.47–2.19, *P* < 0.001) and a 1.90-fold increase in cardiovascular mortality risk (HR = 1.90, 95% CI 1.32–2.75, *P* < 0.001) compared to those in the lower SII group.

Adjustments in Model 2 for demographic factors such as age, sex, ethnicity, and education level did not diminish the statistical significance of these associations. Further adjustments in Model 3 for waist circumference, PIR, smoking status, health insurance, hypertension and diabetes, alongside demographic factors, continued to show that higher SII levels were positively associated with increased risks of mortality. Specifically, every 100-unit increase in SII as a continuous variable was associated with a 4% increase in the risk for both all-cause and cardiovascular mortality. Categorically, individuals in the higher SII group exhibited 1.48 and 1.51 times the risk of all-cause and cardiovascular mortality, respectively, compared to those in the lower SII group, as detailed in Table 2.

**Table 2:** Multivariable Cox regression analysis of association of SII with mortality risk in adults with RA.

	Model 1		Model 2		Model 3	
	HR (95% CI)	*P* value	HR (95% CI)	*P* value	HR (95% CI)	*P* value
All-cause mortality						
SII (continuous)*	1.03 (1.01, 1.05)	< 0.001	1.04 (1.02, 1.05)	< 0.001	1.04 (1.02, 1.05)	< 0.001
Lower SII	Reference		Reference		Reference	
Higher SII	1.80 (1.47, 2.19)	< 0.001	1.50 (1.22, 1.85)	< 0.001	1.48 (1.21, 1.81)	< 0.001
Cardiovascular mortality						
SII (continuous)*	1.03 (1.01, 1.06)	< 0.001	1.04 (1.02, 1.07)	< 0.001	1.04 (1.02, 1.07)	< 0.001
Lower SII	Reference		Reference		Reference	
Higher SII	1.90 (1.32, 2.75)	< 0.001	1.56 (1.06, 2.27)	0.020	1.51 (1.04, 2.18)	0.030

Model 1: Unadjusted;

Model 2: Adjusted age, gender, ethnicity, and education;

Model 3: Adjusted age, gender, ethnicity, education, waist circumference, PIR, smoking status, health insurance, hypertension, and diabetes.

*SII per 100.

RA: Rheumatoid arthritis; SII: Systemic immune-inflammation index; HR: Hazard ratio; CI: Confidence interval.

PIR: Poverty income ratio.

**Figure 3:**
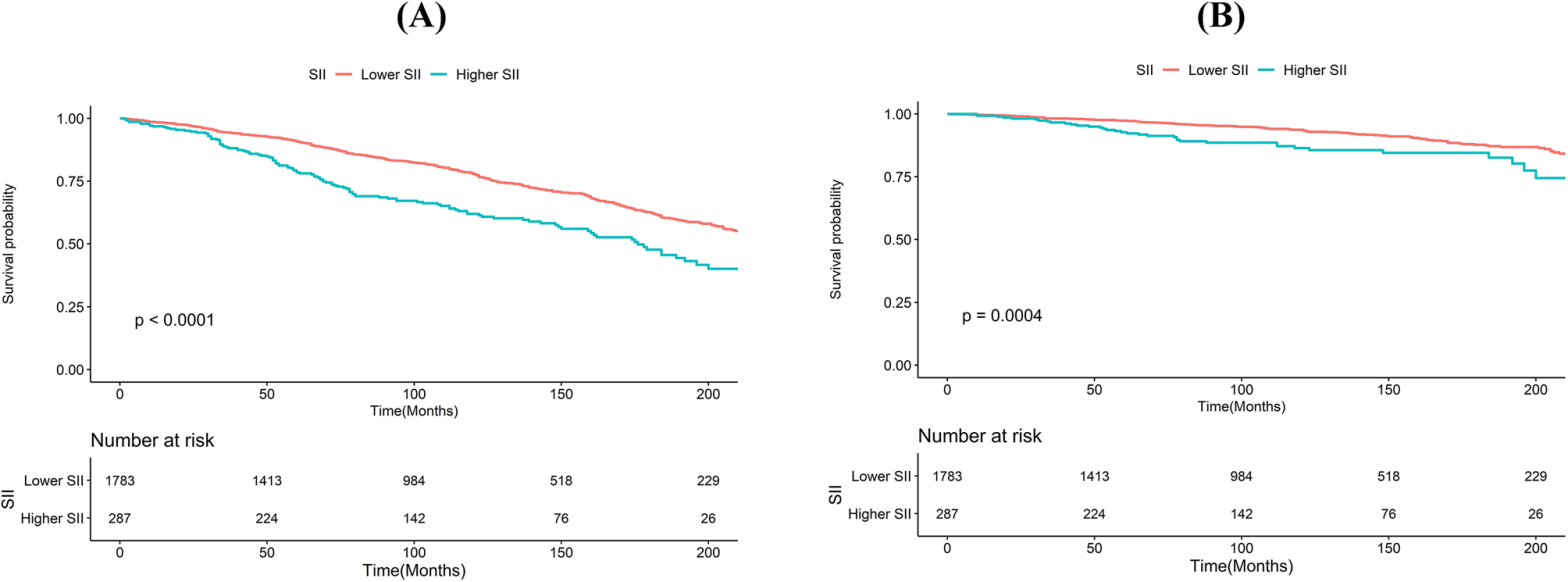
Kaplan–Meier plots for all-cause **(A)** and cardiovascular mortality **(B)** in individuals with RA.

Kaplan–Meier analyses underscored significantly lower survival rates for the higher SII group compared to the lower SII group for both all-cause and cardiovascular mortality (*P*_all-cause mortality_ < 0.0001 and *P*_cardiovascular mortality_ = 0.0004), as depicted in Fig. 3. These results underscore that higher SII levels, regardless of being evaluated as a continuous or categorical variable, consistently and independently predict increased risks of all-cause and cardiovascular mortality among U.S. adults with RA.

### Stratified analyses

**Figure 4:**
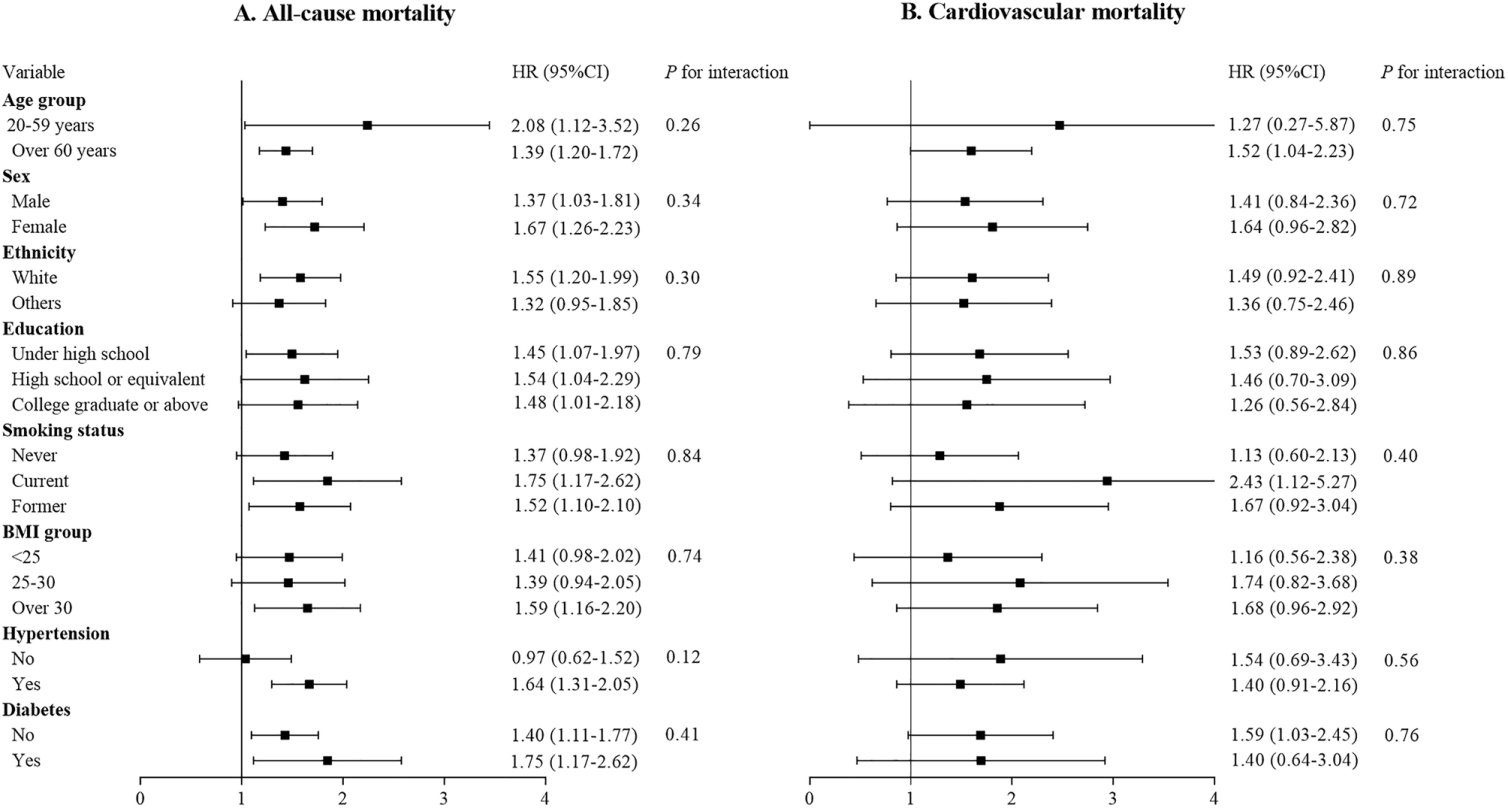
Forest plot of subgroup analyses of SII and mortality risk in RA.

In subgroup analyses that were delineated by factors such as sex, age, race, education level, BMI, and smoking status, a consistent association was observed between elevated SII levels and an increased risk of both all-cause and cardiovascular mortality across all examined subgroups. The analysis revealed no significant interaction effects for all-cause mortality across various subgroups, with *P*-values indicating a lack of statistical significance for sex (*P* = 0.34), age (*P* = 0.26), ethnicity (*P* = 0.30), education level (*P* = 0.79), BMI (*P* = 0.74), smoking status (*P* = 0.84), hypertension (*P* = 0.12), and diabetes (*P* = 0.41). Similarly, for cardiovascular mortality, interaction tests also showed no significant differences by sex (*P* = 0.72), age (*P* = 0.75), ethnicity (*P* = 0.89), education level (*P* = 0.86), BMI (*P* = 0.38), smoking status (*P* = 0.40), hypertension (*P* = 0.56), and diabetes (*P* = 0.76). These findings suggest that the link between higher SII levels and elevated mortality risks remains robust across various demographic and lifestyle subgroups, underscoring the broad applicability of SII as a risk indicator. A forest plot of subgroup analyses is presented in Fig. 4.

### Nonlinear relationship of SII and mortality

**Figure 5:**
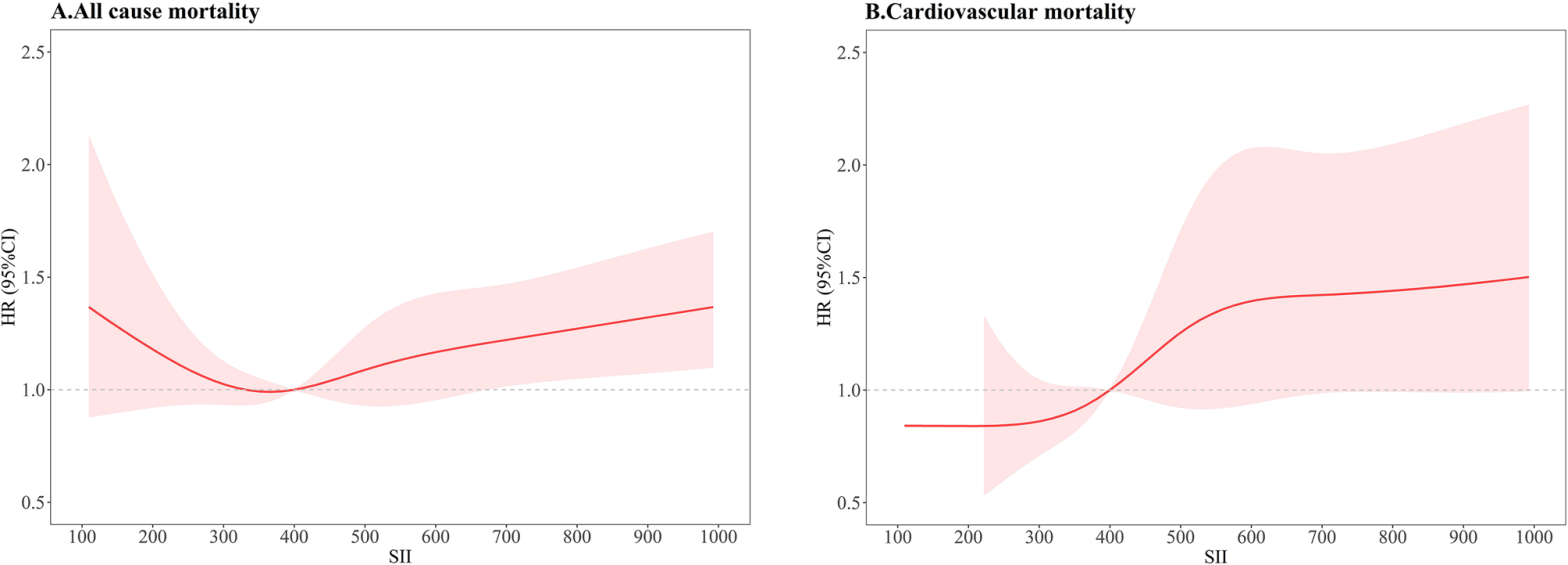
RCS analysis depicting the relationship between SII and all-cause mortality **(A)** as well as cardiovascular mortality **(B)** in participants with RA.

Figure 5A illustrates the RCS curve depicting the association between the SII as a continuous variable and the risk of all-cause mortality among participants with RA. This association was found to be statistically significant (*P*_overall_ < 0.0001), revealing a U -shaped nonlinear relationship (*P*_non-linear_ = 0.90). Conversely, Fig. 5B presents the RCS curve for the association between continuous SII and cardiovascular mortality risk among RA participants. Here, the relationship also appeared nonlinear (*P*_non-linear_ = 0.09), with an increase in SII correlating with a higher risk of cardiovascular mortality. This association was found to be also statistically significant (*P*_overall_ = 0.0046). For cardiovascular deaths, we observed an increase in HR up to an SII of around 600, followed by a leveling off. This finding is consistent with the work by Satis et al.^20^, highlighting the robustness of our findings. Additionally, When SII < 400, the HR < 1, indicating a potential protective effect of lower SII levels on cardiovascular risk. This finding aligns with existing literature suggesting that low inflammation levels may play a protective role in certain contexts. Further investigation into the biological mechanisms underlying this observation is warranted. These analyses underscore distinct relationships between SII levels and different mortality outcomes in individuals with RA, emphasizing the complexity of inflammatory biomarkers in predicting health outcomes.

## Discussion

To our knowledge, this investigation pioneers the exploration into the association between the SII and occurrences of mortality from any cause as well as from cardiovascular conditions among the RA-afflicted adult population of America. Analyzing a comprehensive and demographically reflective cohort of American adults, our research delineates that an augmentation in SII levels distinctly forecasts an escalated risk for mortality, encompassing both all-cause and cardiovascular-specific deaths. The application of Kaplan–Meier analysis techniques elucidated a notably reduced survival probability for subjects exhibiting increased SII values. Moreover, the employment of RCS analysis authenticated a consistent and nonlinear augmentation in mortality risk corresponding with rising SII levels, covering both all-cause and cardiovascular-related mortality. These revelations accentuate the efficacy of SII as an economical, easily obtainable prognostic tool for risk segmentation and prediction within the RA patient demographic, bearing significant ramifications for their clinical oversight.

The scholarly exploration into the relationship between the SII and RA remains limited. However, existing studies indicate that SII scores can serve as effective markers, reflecting RA activity, joint deterioration, and radiographic progression and potentially leading to more precise diagnostic practices^19,20^. Concerning the nexus between SII and RA disease dynamism, the majority of research underscores a positive association, particularly highlighting the significant link between SII values and the treatment effectiveness of TNF-α inhibitors in managing RA. This underscores the pivotal role of SII in gauging the success of such therapeutic interventions.

Numerous investigations have delved into the correlation between the SII and autoimmune disorders. Studies revealed that SII levels escalate significantly in psoriatic arthritis (PsA) patients experiencing moderate to severe disease activity^21,22^. In a similar vein, study of ankylosing spondylitis (AS) demonstrated that an increase in SII is associated with disease severity, suggesting its potential as an innovative metric for monitoring disease severity^24^. Moreover, the implications of SII in predicting adverse pregnancy outcomes among expectant mothers afflicted with systemic lupus erythematosus (SLE) indicated its predictive utility in such scenarios^25^, as well as in forecasting SLE and indicating nephritis^26^. Regarding Behçet disease (BD), research has highlighted the potential of the SII as an adjunctive tool for assessing disease status. It is recommended that physicians exercise heightened vigilance for patients with higher levels of SII during the initial evaluation^27^.

Existing literature has highlighted a link between elevated SII levels and an increased risk of mortality both in the general population and among individuals with conditions characterized by significant inflammatory processes^28–31^. Regarding the acute ischemic stroke in intensive care units, one study identified a positive, albeit non-linear, relationship between SII and in-hospital mortality^11^, as well as in individuals with stroke-associated pneumonia^32^. Furthermore, research has shown that SII is associated with a heightened risk of mortality in adults with asthma^33^. Within oncology, studies established SII as an independent prognostic factor for overall mortality, underscoring its potential utility as a prognostic marker^34,35^. In cardiac surgery, a meta-analysis involving over 3245 patients revealed that a high preoperative SII significantly predicts an increased risk of postoperative atrial fibrillation, suggesting its value as a predictive biomarker^36^.

Despite the growing body of research linking the SII to mortality risks in various diseases, its prognostic value for survival in RA patients has not been extensively studied. Our research fills this critical void, presenting novel insights that establish the SII as a standalone forecaster of both all-cause and cardiovascular mortality in individuals with RA. Additionally, our study suggests that the formulation of patient-specific SII thresholds could enhance the marker's predictive precision in a clinical milieu. In comparison to the mortality risks of cerebrovascular and cardiovascular conditions, the potential lethality within autoimmune diseases, especially RA, might be underrated. Hence, this domain remains crucial for further inquiry. Our findings reveal that RA patients with an SII beyond a certain limit face a significantly elevated risk of all-cause mortality, documented at 43.90% (126 out of 285 individuals). The likelihood of succumbing to cardiovascular complications was also notable, standing at 12.89% (37 out of 285 individuals). This underscores the urgency of prioritizing mortality risk prediction in RA patients, notably through the deployment of readily available and clinically viable biomarkers. The SII emerges from our study as a pivotal index, illustrating its prospective value in the prognostication and risk categorization of RA patients within clinical practices, thereby reinforcing the importance of incorporating this biomarker into routine patient evaluations and management strategies.

The complex pathophysiological mechanisms at the core of autoimmune disorders, including the dynamic between the body's innate and adaptive immune systems, have been extensively studied, shedding light on the intricate workings of diseases like RA. The SII, which aggregates counts of neutrophils, platelets, and lymphocytes, offers a prism through which the inflammatory state and immune responsiveness of the body can be viewed. A heightened neutrophil count often signals an active, unspecific inflammatory reaction, whereas a diminished lymphocyte count may reflect a compromised immune defense^37^. Evidence consistently points to the disordered regulation of innate immunity and inflammation as pivotal in the etiology of RA^38,39^. Neutrophils, serving as frontline responders in inflammation and immune activation within RA^39^, also assume roles as antigen-presenting cells, aiding in the perpetuation of chronic inflammation and autoimmune processes by presenting antigens and activating T cells^40^. Moreover, neutrophils release proteases like elastase, contributing to the degradation of cartilage and bone in RA. In the realm of autoimmune diseases, a surge in platelet count typically acts as a reaction to ongoing inflammation, indicative of the body's aberrant assault on its own tissues. Conditions such as RA, SLE, and inflammatory bowel disease provoke an elevated inflammatory response, influencing platelet numbers. This increase in platelets, therefore, might be seen as the body's attempt to counteract inflammation or infection. An escalated SII denotes not only an increased neutrophil activation and innate inflammatory response but also suggests a key role in the pathogenesis and progression of RA. The heightened systemic inflammation, as indicated by a raised SII, may further expedite atherosclerotic processes, elevating cardiovascular risk and mortality^41^. On the flip side, lymphocytes, including regulatory T cells, are integral in dampening inflammatory reactions and reinstating immune equilibrium^42^. A reduced lymphocyte tally, as implied by a high SII, could signify a deficiency in anti-inflammatory immunity.

## Limitations

Our study comes with several limitations that merit attention. Firstly, the observational nature of the research restricts our ability to draw causal conclusions. While we have established associations between the SII and various outcomes, establishing a direct cause-and-effect relationship remains beyond our reach. Secondly, despite our efforts to account for a wide array of potential confounders, the possibility of residual confounding cannot be entirely ruled out. Factors not measured in our study, such as specific comorbidities, the intensity of smoking habits, medication usage, and particular characteristics of RA, might have a bearing on the associations we observed. Thirdly, the measurement of SII was conducted at a single point in time, which does not account for potential fluctuations over time or in response to treatment interventions. A series of SII measurements over time might offer a more comprehensive understanding of the inflammatory process. Fourthly, the diagnosis of RA is based on the self-reported questionnaires, which may cause selection bias. Lastly, the focus on a U.S. adult population limits the broad applicability of our findings to other demographic groups. To confirm the universality of our results, further research involving varied populations is necessary. Despite these limitations, the study's strengths lie in its large, nationally representative sample size, extended duration of follow-up, and the thoroughness of the adjustments made for confounding variables, all of which contribute to the robustness and reliability of our findings.

## Conclusion

In conclusion, the SII offers a straightforward indicator of the equilibrium between detrimental innate inflammation and beneficial adaptive immunity. Our investigation, utilizing a comprehensive and nationally representative sample, reveals that elevated SII levels independently forecast a greater risk of mortality from all causes, as well as cardiovascular-specific mortality, in individuals suffering from RA. These insights underscore the clinical relevance of the SII as an affordable and readily accessible biomarker. Its incorporation into regular clinical practice could significantly enhance the precision of risk assessment and forecasting for patients with RA, facilitating more tailored and effective management strategies. Specifically, patients with high SII levels could be identified for more stringent cardiovascular risk management, including closer monitoring, lifestyle interventions, and aggressive pharmacological treatments to mitigate their increased risk of mortality.

### Supplementary Information

Supplementary Information.

## Data Availability

The study made use of datasets that are available to the public. The National Health and Nutrition Examination Survey dataset can be accessed at https://www.cdc.gov/nchs/nhanes/index.htm. The dataset used to analyze could be found in Supplementary Material.
